# Identification and Evaluation of Novel Protective Antigens for the Development of a Candidate Tuberculosis Subunit Vaccine

**DOI:** 10.1128/IAI.00014-18

**Published:** 2018-06-21

**Authors:** Elena Stylianou, Rachel Harrington-Kandt, Julia Beglov, Naomi Bull, Nawamin Pinpathomrat, Gwendolyn M. Swarbrick, Deborah A. Lewinsohn, David M. Lewinsohn, Helen McShane

**Affiliations:** a The Jenner Institute, University of Oxford, Oxford, United Kingdom; b Department of Pediatrics, Oregon Health & Sciences University, Portland, Oregon, USA; c Department of Molecular Microbiology and Immunology, Oregon Health & Sciences University, Portland, Oregon, USA; d Department of Medicine, Oregon Health & Sciences University, Portland, Oregon, USA; Weill Cornell Medical College

**Keywords:** Mycobacterium tuberculosis, tuberculosis, vaccines, viral vectors

## Abstract

The development of a vaccine against tuberculosis (TB), a disease caused by Mycobacterium tuberculosis, is urgently needed. The only currently available vaccine, M. bovis BCG, has variable efficacy. One approach in the global vaccine development effort is focused on boosting BCG using subunit vaccines. The identification of novel antigens for inclusion in subunit vaccines is a critical step in the TB vaccine development pathway. We selected four novel mycobacterial antigens recognized during the course of human infection. A replication-deficient chimpanzee adenovirus (ChAdOx1) was constructed to express each antigen individually, and these vectors were evaluated for protective efficacy in murine M. tuberculosis challenge experiments. One antigen, PPE15 (Rv1039c), conferred significant and reproducible protection when administered alone and as a boost to BCG vaccination. We identified immunodominant epitopes to define the protective immune responses using tetramers and intravascular staining. Lung parenchymal CD4^+^ and CD8^+^ CXCR3^+^ KLRG1^−^ T cells, previously associated with protection against M. tuberculosis, were enriched in the vaccinated groups compared to the control groups. Further work to evaluate the protective efficacy of PPE15 in more stringent preclinical animal models, together with the identification of further novel protective antigens using this selection strategy, is now merited.

## INTRODUCTION

Tuberculosis (TB) is the deadliest infectious disease, with 10.4 million new cases and 1.7 million deaths occurring in 2016 (1). Mycobacterium bovis bacillus Calmette-Guérin (BCG) is the only available vaccine against TB. Despite variable efficacy throughout the world, it is widely used because of its effectiveness against childhood forms of TB (2, 3). As TB in adolescents and adults is responsible for much of the disease and transmission burden, an effective vaccine that targets this population would have a significant effect on reducing the incidence of this disease.

The TB vaccine field is developing vaccines to either replace or boost neonatal BCG vaccination in adolescents (4). The booster vaccines are typically subunit vaccines, composed of one or more mycobacterial antigens delivered either as DNA, as proteins in adjuvant, or as recombinant viral vectors. Sequencing of the genome of Mycobacterium tuberculosis, the causative agent of TB disease, revealed that it encodes about 4,000 genes (5), which subsequently led to efforts to identify protective antigens for vaccine development (6). Selecting the best antigens to include in subunit vaccines presents a major challenge, in part due to the lack of immune correlates of protection. In addition, there is limited knowledge about the function of a large number of antigens, their differential expression at different stages of the mycobacterium life cycle (7), and the relative importance of secreted versus nonsecreted antigens (8), complicating the selection further. A recent study looking at the sequence variation of human T cell epitopes revealed that the majority of mycobacterial antigens and epitopes are hyperconserved, despite the presence of T-cell responses against the bacterium during the course of M. tuberculosis infection (9). Whether this hyperconservation results in immune responses that favor the bacterium (9) or whether these antigens have an important function (10) remains to be clarified. However, if the first possibility is true, selecting immunodominant antigens for vaccine development might not be the best strategy, and the reverse would be the case if the latter possibility is true.

Only 11 mycobacterial antigens have progressed to human clinical trials as part of subunit vaccines, with antigens from the antigen 85 (Ag85) complex being the most commonly used. The protein-adjuvanted vaccines that are currently being evaluated in clinical trials utilize a greater diversity of mycobacterial antigens than virus-vectored vaccines. Adjuvanted protein vaccine candidates use 11 mycobacterial antigens in different combinations (PepA, PPE18, Ag85A, Ag85B, TB10.4, PPE42, EsxV, EsxW, Rv1813, ESAT6, Rv2660c) (11–15), whereas only 3 antigens have been evaluated as recombinant viral vectors (Ag85A, Ag85B, TB10.4) (16–18).

In order to identify novel protective mycobacterial antigens as well as to exploit the potency of viral vectors, we screened a number of potential candidate antigens that had been previously identified on the basis of their recognition by CD8^+^ T cells from patients with active TB disease and latent M. tuberculosis infection (19, 20). Major histocompatibility complex (MHC) class II-restricted CD4^+^ T cells are essential for protection against M. tuberculosis (21–23); however, the importance of CD8^+^ T cells is less defined. It is clear that this T cell subset plays an important role in M. tuberculosis control, as β_2_ microglobulin-, MHC class I-, and CD8^+^-deficient mice are all more susceptible to M. tuberculosis infection than wild-type controls (23–25). The importance of MHC class I-restricted CD8^+^ T cells is further emphasized in nonhuman primate studies (26, 27). These studies highlight an important role for CD8^+^ T cells in controlling M. tuberculosis infection and the need to develop vaccine strategies that incorporate their protective potential.

Using immune parameters that have been associated with mycobacterial protection (28), we selected four human immunodominant antigens to evaluate as subunit vaccines. A chimpanzee adenoviral vector was modified to express each of the antigens individually (29, 30). Chimpanzee adenoviruses are ideal vaccine delivery platforms, as they have the ability to induce strong humoral and cellular responses, without being compromised by preexisting antivector immunity (29, 31–33). Furthermore, they are a potent method of inducing CD8^+^ T cell responses, and thus, this antigen delivery system would allow us to exploit the human CD8^+^ T cell epitopes within the antigens selected. The importance of CD4^+^ T cells in controlling infection is critical, and all antigens selected also induced CD4^+^ T cell responses.

We found that one of the four antigens evaluated, PPE15, induced a reproducible protective immune response. Further analysis of the immune responses induced by PPE15 identified T cell subsets that were associated with protection.

## RESULTS

### Antigen identification.

For antigens used in vaccines designed for human administration, recognition by human cells is critical. For this reason, 15 novel antigens that were initially shown to be strongly and commonly recognized by CD8^+^ T cells from TB patients and individuals with latent M. tuberculosis infection (LTBI) in Portland, OR, and Uganda were selected (19, 20). All the antigens selected were also associated with robust CD4^+^ T cell stimulation in subjects with active TB and LTBI (D. A. Lewinsohn, unpublished data).

The absence of a human M. tuberculosis challenge model requires all new vaccine candidates to be first assessed in the mouse model before proceeding to more stringent animal models, such as the guinea pig and nonhuman primate models. To confirm that these antigens were also broadly and commonly recognized during the course of murine M. tuberculosis infection, we infected BALB/c and C57BL/6 mice, two of the most common strains used in TB vaccine studies, with M. tuberculosis (see Fig. S1 in the supplemental material). Cells from lungs and spleens were stimulated with complete or partial peptide pools, and the percentage of antigen-specific cells releasing the Th1 cytokines gamma interferon (IFN-γ), tumor necrosis factor alpha (TNF-α), and interleukin-2 (IL-2) was measured (Fig. S1A).

All 15 antigens were recognized, but they were recognized at different levels, and recognition varied between mouse strains and organs (Fig. S1B to I). Antigens were then ranked on the basis of the potency of induction of immune responses known to be associated with protection: IFN-γ secretion by CD4^+^ and CD8^+^ T cells and the induction of polyfunctional (IFN-γ, TNF-α, IL-2) T cells in both the lung and spleen (Fig. S2) (34–36). Using the above-described data in combination with the human recognition data, we selected four lead antigens for evaluation of protective efficacy. Three, PPE15 (Rv1039c), PPE51 (Rv3136), and PE12 (Rv1172c), were all selected for their overall good recognition by CD4^+^ and CD8^+^ T cells in the lungs and spleens of both strains of mice, as well as their ability to induce polyfunctional T cells. The fourth, PE3 (Rv0159c), although weakly recognized by BALB/c mice (Fig. S1B to E), was strongly recognized by C57BL/6 mice (Fig. S1F to I).

### Antigen-specific responses after BCG vaccination.

The aim of a subunit TB vaccine is to boost the protective efficacy of BCG. We therefore confirmed the presence of the selected antigens in BCG Pasteur using the NCBI nucleotide search tool. All antigens were more than 99.7% similar between M. tuberculosis Erdman and BCG Pasteur.

**FIG 1:**
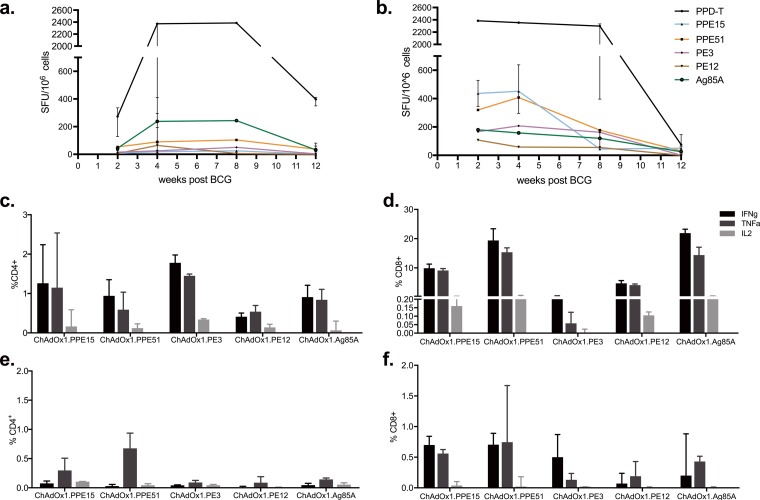
Antigen-specific responses after BCG and ChAdOx1 immunizations. (a and b) Splenocytes from BCG-vaccinated BALB/c (a) and C57BL/6 (b) mice were stimulated with PPE51, PPE15, PE3, or PE12, and IFN-γ production was measured at different time points postvaccination using an ELISpot assay. Stimulations with Ag85A and PPD-T were used as positive controls. Each symbol represents the median response (in number of spot-forming units [SFU]/10^6^ cells) for three animals, and the error bars represent the range of values. (c to f) CB6F1 mice were vaccinated with a single intranasal administration of ChAdOx1 expressing the four selected antigens; the percentage of antigen-specific CD4^+^ or CD8^+^ T cells expressing IFN-γ, TNF-α, and IL-2 was measured in the lungs (c and d) and spleen (e and f). The bars represent the median response of five animals, and error bars indicate the interquartile range. Data are representative of those from two independent experiments.

In order to evaluate the level of PPE15-, PPE51-, PE3-, and PE12-specific T cells induced by BCG vaccination, BALB/c and C57BL/6 mice were vaccinated with intradermal (i.d.) BCG, and mice were harvested at 2, 4, 8, and 12 weeks postvaccination to measure antigen-specific IFN-γ responses (Fig. 1a and b).

Purified protein derivative of M. tuberculosis (PPD-T) responses peaked after 2 weeks in C57BL/6 mice, whereas they peaked at 4 weeks in BALB/c mice (Fig. 1b and a). The Ag85A peptide pool was used as a control antigen. In BALB/c mice, the responses to Ag85A were the highest, whereas the responses to the four candidate antigens were low at all time points tested (Fig. 1a). The responses to all antigens decreased by week 12. In BCG-vaccinated C57BL/6 mice, the level of the Ag85A-specific response was similar to that in BALB/c mice, whereas the responses to PPE15 and PPE51 were much higher (Fig. 1b). These responses were detected soon after vaccination at 2 weeks and were retained at high levels up to week 4, after which there was a progressive decrease for the later time points. Responses to PE3 and PE12 were also detectable but were weaker.

### Evaluation of the immunogenicity of recombinant chimpanzee adenoviral vectors expressing novel antigens.

In order to assess the vaccine potential of these antigens, a simian chimpanzee adenovirus (ChAdOx1) was modified to express either PPE15, PPE51, PE3, or PE12 (ChAdOx1.PPE15, ChAdOx1.PPE51, ChAdOx1.PE3, or ChAdOx1.PE12, respectively). ChAdOx1 expressing Ag85A (ChAdOx1.85A) was used as a comparative vaccine candidate for its ability to induce strong cell-mediated immune responses (30). To allow for more diversity in MHC restriction, CB6F1/Crl (CB6F1) mice were used for immunizations.

Mice were vaccinated with a single intranasal (i.n.) administration of the different viruses and harvested 2 weeks later for evaluation of antigen-specific responses in the lung and spleen (Fig. 1c to f).

In the lung, all viruses induced antigen-specific CD4^+^ T cells secreting all three cytokines (Fig. 1c). The percentage of antigen-specific CD8^+^ T cells secreting Th1 cytokines was higher than that for CD4^+^ T cells (Fig. 1d). All viruses except ChAdOx1.PE3 induced a high percentage of CD8^+^ T cells secreting IFN-γ and TNF-α; however, a partial peptide pool was used for *ex vivo* stimulation for this antigen at the time. Repetition of immunogenicity with the full peptide pool indicated that ChAdOx1.PE3 was also able to induce strong CD8^+^ T cell responses (Fig. S3). Spleen responses for both CD4^+^ and CD8^+^ cells were much lower (Fig. 1e and f).

### Evaluation of protective efficacy of ChAdOx1 expressing novel antigens.

**FIG 2:**
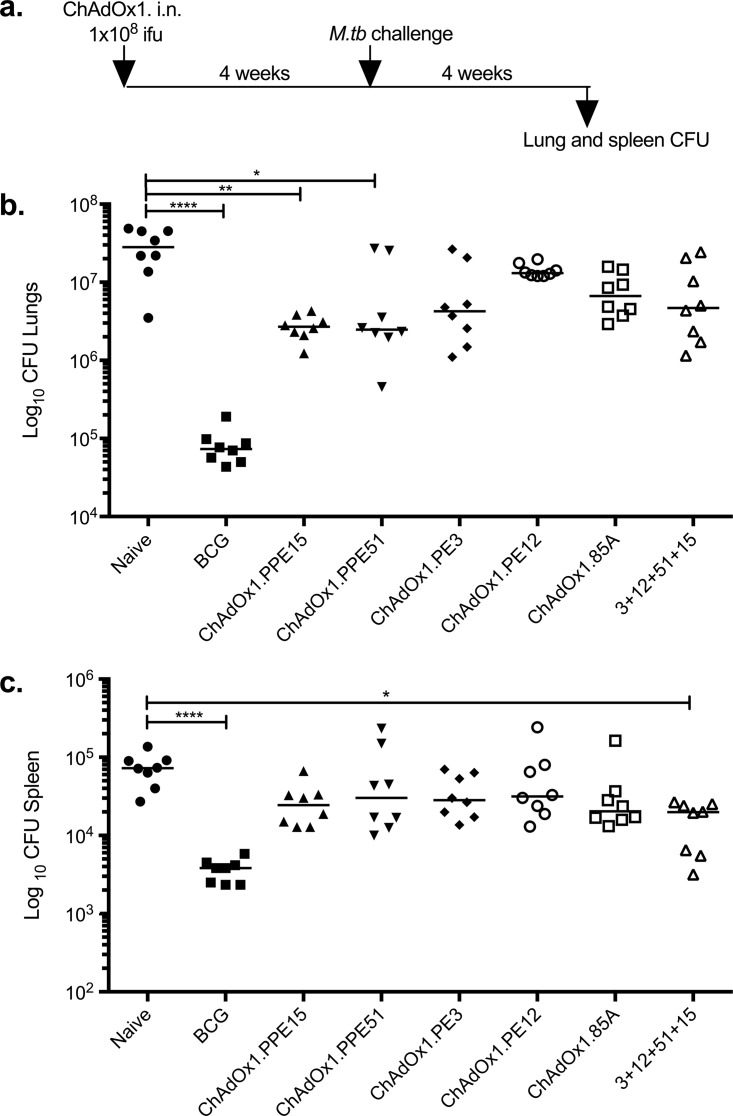
Testing the protective efficacy of the four modified ChAdOx1 vaccine candidates against M. tuberculosis infection. (a) Experimental schema. ifu, infectious units; CFU, colony-forming units. (b and c) Lung (b) and spleen (c) bacterial loads of naive and vaccinated CB6F1 mice after aerosol M. tuberculosis Erdman infection. Vaccinated mice received a single i.n. ChAdOx1 vaccination. 3+12+51+15 represents a group of animals that received the four viruses in one administration (*n* = 8 mice). Representative data from one of two duplicate experiments are shown. Each symbol represents one animal, and the lines represent the median for each group. The Kruskal-Wallis test followed by Dunn's multiple-comparison test was used to test for significant differences. *, *P* < 0.05; **, *P* < 0.01; ****, *P* < 0.0001.

To investigate the protective potential of these viruses, mice were vaccinated with a single intranasal dose of each virus and challenged with aerosolized M. tuberculosis 4 weeks later (Fig. 2). A group of mice received all four viruses in combination to test whether there would be an additive synergistic protective effect. This group received the same dose of each virus preparation as the remaining groups and, therefore, four times the number of viral particles received by the rest of the mice. A positive-control group received BCG and was allowed to rest for 6 weeks before challenge (Fig. 2a). All mice were harvested at 4 weeks postchallenge for enumeration of the bacterial colony-forming units (CFU) in the lungs and spleen (Fig. 2b and c).

The BCG-vaccinated group had a lung bacterial load 2.5-log_10_ CFU lower (Kruskal-Wallis test, *P* < 0.0001; Dunn's multiple-comparison test, *P* < 0.0001) than the naive control group (Fig. 2b). Vaccination with ChAdOx1.PPE15 and ChAdOx1.PPE51 also significantly reduced the lung bacterial load compared to that in the unvaccinated group (Kruskal-Wallis test, *P* < 0.0001; Dunn's multiple-comparison test, *P* < 0.01 and *P* < 0.05, respectively). The ChAdOx1.PE3-, ChAdOx1.PE12-, and ChAdOx1.85A-vaccinated groups did not have significantly lower bacterial loads than the naive group. Administering all four viruses simultaneously did not result in an additive protective effect.

In the spleen, the BCG-vaccinated group had bacterial loads 1.5 log_10_ CFU lower than those in the naive group (Kruskal-Wallis test, *P* < 0.0001; Dunn's multiple-comparison test, *P* < 0.0001) (Fig. 2c). The group vaccinated with all four viruses simultaneously had a significantly lower bacterial load than the unvaccinated control group (Kruskal-Wallis test, *P* < 0.0001; Dunn's multiple-comparison test, *P* < 0.05), with three mice being protected to a level similar to that for mice in the BCG-vaccinated group.

**FIG 3:**
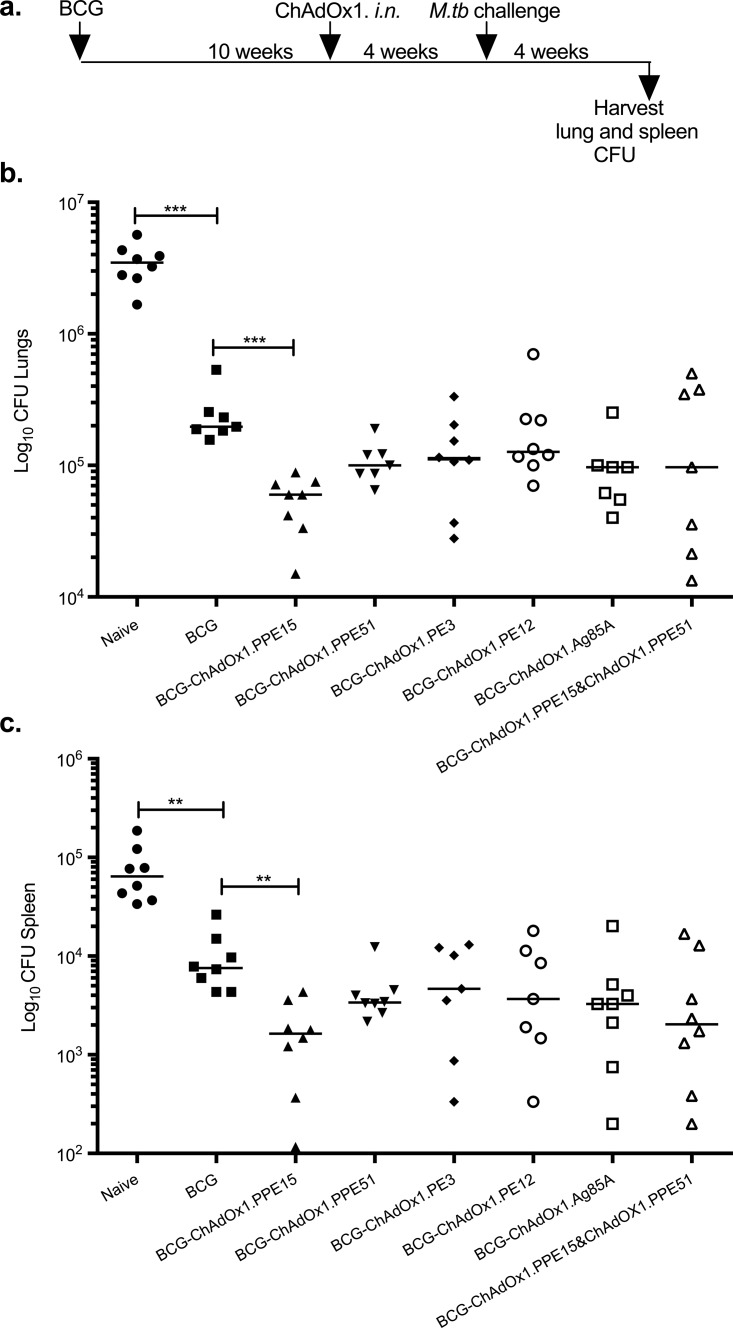
Assessing the ability of ChAdOx1 candidate vaccines to improve the protective efficacy of BCG. (a) Experimental schema used to immunize CB6F1 mice. (b and c) Lung (b) and spleen (c) bacterial loads were measured 4 weeks after aerosol M. tuberculosis infection. Each symbol represents one animal, and the lines represent the median for each group (*n* = 8 mice). The Kruskal-Wallis test followed by Dunn's multiple-comparison test was used to test for significant differences. **, *P* < 0.01; ***, *P* < 0.001. The findings were confirmed by five independent experiments.

As these vaccines are intended for use as a boost to previous BCG vaccination, it was important to investigate whether the protective efficacy provided by a prior BCG vaccination could be further improved by the novel antigens. CB6F1 mice were primed with i.d. BCG and boosted with intranasal ChAdOx1. Ten weeks later (Fig. 3a). ChAdOx1.85A was used as a control antigen. ChAdOx1.PPE15 and ChAdOx1.PPE51, which provided the best protection in the previous experiments, were mixed in equal amounts and used to vaccinate a group of mice to assess whether there was an additive protective effect. At 4 weeks postchallenge, mice were harvested for lung and spleen bacterial load enumeration (Fig. 3a).

In the lung, BCG vaccination significantly reduced the bacterial load compared to that in the unvaccinated control group (*P* < 0.001) (Fig. 3b). Boosting of BCG with intranasal ChAdOx1.PPE15 further improved BCG protection by 0.52 log_10_ (Kruskal-Wallis test, *P* < 0.01; Dunn's multiple-comparison test, *P* < 0.01), whereas no significant improvement in protection was observed with the other constructs. Mixing ChAdOx1.PPE15 and ChAdOx1.PPE51 resulted in high variability in the bacterial load within the group and no improvement in protection.

In the spleen, BCG-vaccinated mice had significantly lower numbers of CFU than mice in the unvaccinated control group (*P* < 0.01) (Fig. 3c). As in the lung, boosting with ChAdOx1.PPE15 improved BCG protection by 0.7 log_10_ in the spleen (Kruskal-Wallis test, *P* < 0.0455; Dunn's multiple-comparison test, *P* < 0.01). None of the remaining vaccines improved the protection provided by BCG.

### Identifying patterns of recognition for protective versus nonprotective antigens.

Of the four antigens selected, PPE15 was the only one that provided consistent protection and PPE51 provided intermediate protection. In contrast, PE12 and PE3 provided little to no protection. Epitope mapping experiments were performed to identify potential differences in immune recognition between the four antigens as well as in the breadth and strength of these immune responses that could help explain the observed differences in protection. An IFN-γ-specific enzyme-linked immunosorbent spot (ELISpot) assay was used to identify immunogenic epitopes, and flow cytometry was subsequently used to discriminate CD4^+^ or CD8^+^ T cell cytokine production. Epitope mapping was performed using spleen tissue samples from BALB/c and C57BL/6 mice vaccinated with i.d. ChAdOx1.PPE15, ChAdOx1.PPE51, ChAdOx1.PE3, or ChAdOx1.PE12.

**FIG 4:**
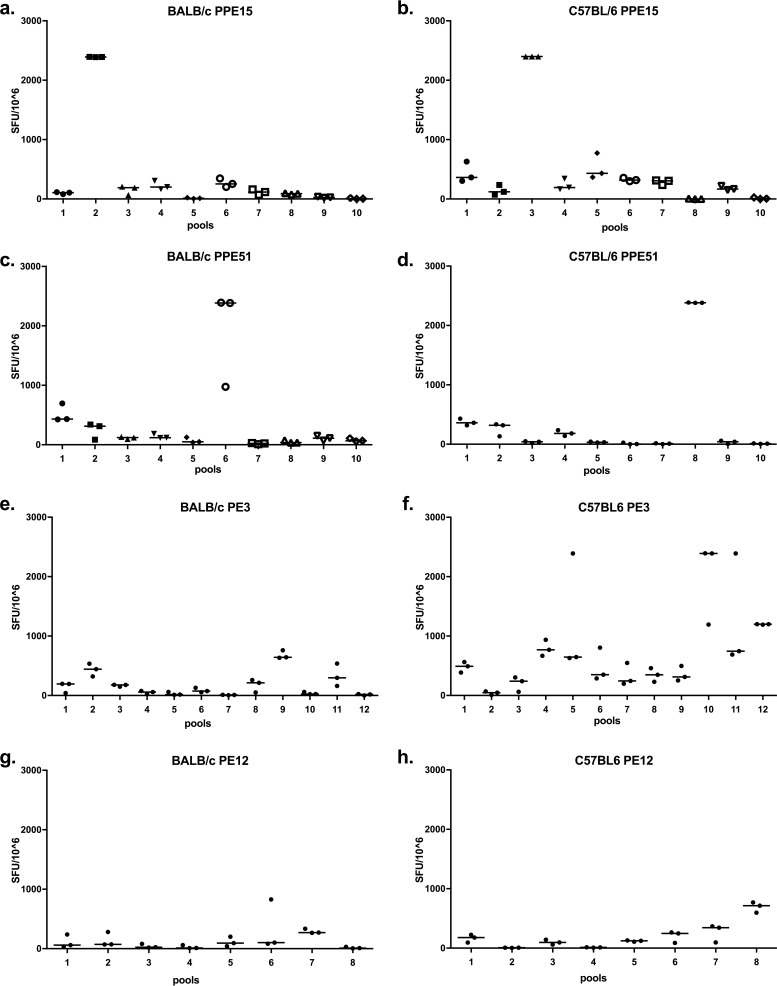
Measurement of the breadth and strength of the immune response induced after administration of the different ChAdOx1 vaccines. The antigen-specific IFN-γ immune responses in BALB/c and C57BL/6 mice were measured at 2 weeks postimmunization. (a and b) ChAdOx1.PPE15 vaccination; (c and d) ChAdOx1.PPE51 vaccination; (e and f) ChAdOx1.PE3 vaccination; (g and h) ChAdOx1.PE12 vaccination. Splenocytes from three vaccinated animals were stimulated with pools of 10 15-mer peptides overlapping by 11 amino acids. Each dot represents one animal, and the lines represent the median response. Data are representative of those from two independent experiments.

Pools of 10 15-mer peptides overlapping by 11 amino acids were prepared and used to stimulate splenocytes from mice vaccinated with ChAdOx1 expressing the different antigens (Fig. 4). Pools 2 and 3 from PPE15 induced the highest responses in BALB/c and C57BL/6 mice, respectively (Fig. 4a and b). The individual peptides of the most immunogenic pools were then used to identify key epitopes (Fig. S4). CB6F1 mice were included in this experiment. The N-terminal part of PPE15 was the most immunogenic, with a total of four dominant epitopes: one in BALB/c mice and three in C57BL/6 mice (Fig. S4a). In BALB/c mice, epitope (2.1/2.2) was recognized by CD8^+^ T cells (Fig. S5a and b), and in C57BL/6 mice, epitope 1.1/1.2 was recognized by CD4^+^ T cells, epitope 3.4 was recognized by both CD4^+^ and CD8^+^ T cells, and epitope 5.8 was recognized by CD8^+^ T cells (Fig. S5c and d).

PPE51 was also well recognized by both mouse strains, with the strongest responses being induced by pools 6 and 8 in BALB/c and C57BL/6 mice, respectively (Fig. 4c and d). Epitope mapping identified three immunodominant epitopes in BALB/c mice and two in C57BL/6 mice (Fig. S4b). In BALB/c mice, all peptides were restricted by CD8^+^ T cells (Fig. S5e and f), and in C57BL/6 mice, peptide 1.1 was restricted by CD4^+^ T cells, whereas the immunodominant peptides in pool 8 were restricted by CD8^+^ T cells (Fig. S5g and h).

ChAdOx1.PE3 induced strong immune responses to more than one peptide pool in C57BL/6 mice, but the responses were weaker in BALB/c mice (Fig. 4e to f). Epitope mapping confirmed the large number of peptides inducing strong responses in C57BL/6 mice and the two immunodominant epitopes in BALB/c mice (Fig. S4c).

Responses to ChAdOx1.PE12 were weak (Fig. 4g and h). In C57BL/6 mice, peptide 4 from pool 8 was mostly recognized, whereas in BALB/c mice, peptide 6 in pool 7 was mostly recognized (Fig. S4d).

### Protective efficacy of ChAdOx1.PPE15 in the two parental mouse strains.

The protective efficacy of ChAdOx1.PPE15 could potentially be due to the broader recognition of the antigen (because of the use of the cross CB6F1 strain) or due to the presence of a potentially protective epitope. To investigate this, the two CB6F1 mouse parental strains were vaccinated with i.n. ChAdOx1.PPE15 and then challenged with M. tuberculosis.

**FIG 5:**
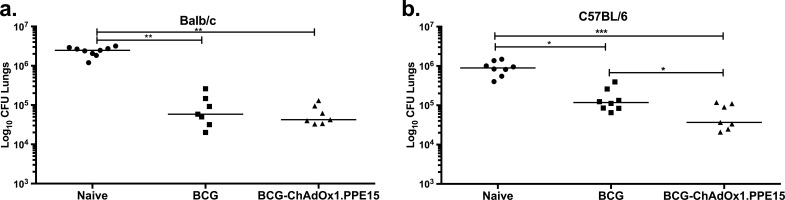
Evaluation of the protective efficacy of BCG-ChAdOx1.PPE15 in C57BL/6 and BALB/c. The lung bacterial loads 4 weeks after aerosol M. tuberculosis infection in BCG-primed BALB/c (a) and C57BL/6 (b) mice are shown. Each symbol represents one animal, and the lines represent the median for each group (*n* = 8 mice). The Kruskal-Wallis test followed by Dunn's multiple-comparison test was used to test for significant differences. *, *P* < 0.05; **, *P* < 0.01; ***, *P* < 0.001.

BALB/c and C57BL/6 mice were primed with BCG and boosted 10 weeks later with i.n. ChAdOx1.PPE15. Four weeks after the last immunization, mice were challenged with aerosolized M. tuberculosis (Fig. 5). ChAdOx1.PPE15 did not improve the protection provided by BCG in BALB/c mice, but it significantly improved the protection provided by BCG in C57BL/6 mice (*P* < 0.05) (Fig. 5a and b).

### Recognition of PPE15 immunodominant epitopes during M. tuberculosis infection.

**FIG 6:**
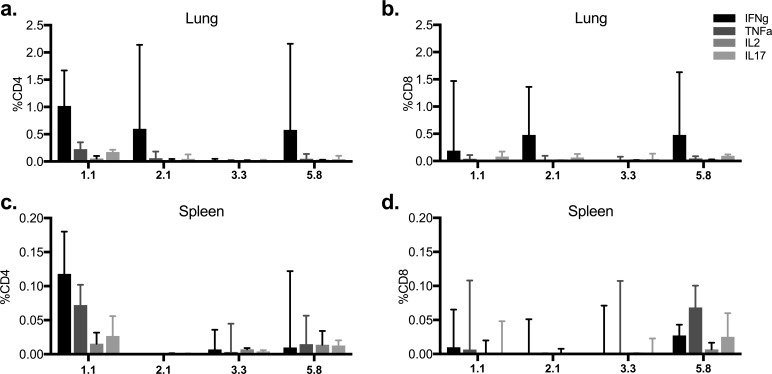
Recognition of the immunodominant epitopes of PPE15 during natural M. tuberculosis infection. CB6F1 mice were challenged with M. tuberculosis, and lung (a and b) and spleen (c and d) cells were collected at 4 weeks postinfection. Cells were stimulated with the immunodominant epitopes, and the percentage of cytokine-secreting CD4^+^ and CD8^+^ T cells was measured. The bars represent the median values for three animals, and the lines above the bars represent the intermediate ranges.

To investigate whether the difference in protection provided by ChAdOx1.PPE15 between the two mouse strains was due to differences in PPE15 epitope recognition following natural infection, lung and spleen cells from infected animals were stimulated with the immunodominant epitopes. The BALB/c mouse epitope (2.1) was as well recognized as the C57BL/6 mouse epitopes in the lung (Fig. 6a and b). However, there was no recognition of the BALB/c mouse epitope in the spleen (Fig. 6c and d).

### Immune responses induced by intranasal ChAdOx1.PPE15 in the lung parenchyma versus lung vasculature.

**FIG 7:**
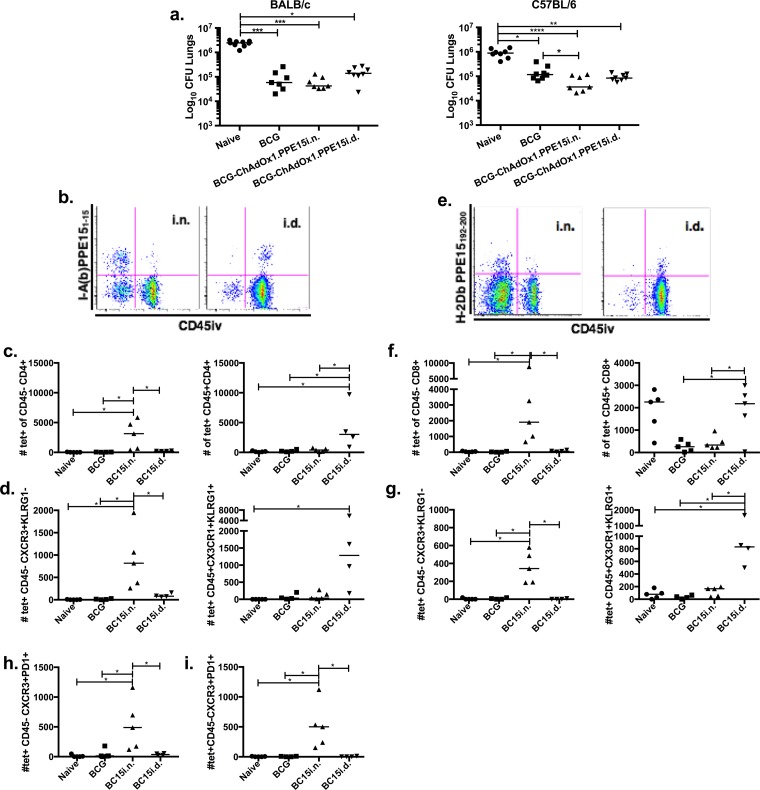
Characterization of the phenotype of antigen-specific T cells in the lung parenchyma and vasculature of ChAdOx1.PPE15-vaccinated mice. (a) BALB/c and C57BL/6 mice were primed with BCG and boosted 10 weeks later with either i.n. or i.d. ChAdOx1.PPE15. At 4 weeks after the last vaccination, the animals were challenged with 50 to 100 CFU of aerosolized M. tuberculosis Erdman. Each dot represents one animal, and the lines represent the median value for each group (*n* = 8 mice). The Kruskal-Wallis test followed by Dunn's multiple-comparison test was used to test for significant differences. *, *P* < 0.05; **, *P* < 0.01; ***, *P* < 0.001; ****, *P* < 0.0001. Tetramer staining was combined with intravascular staining to discriminate the lung parenchyma from lung vasculature cells. (b) Representative plots of I-A(b) PPE15_1–15_ tetramer and intravascular staining of CD4^+^ T cells. (c) Number of tetramer-positive (tet^+^) and intravascular staining-negative CD45^−^ CD4^+^ (left) and intravascular staining-positive CD45^+^ CD4^+^ (right) T cells. (d) Number of tetramer-positive and intravascular staining-negative CD4^+^ T cells that are CXCR3^+^ and KLRG1^−^ (left) and tetramer-positive and intravascular staining-positive CD4^+^ T cells that are CX3CR1^+^ KLRG1^+^ (right). (e to g) The same as for panels b to d, respectively, but for CD8^+^ T cells using the H-2D^b^ PPE15_192–200_ tetramer. (h and i) Number of tetramer-positive and intravascular staining-negative CD4^+^ (h) or CD8^+^ (i) T cells that were CXCR3^+^ PD1^+^. *, *P* < 0.05. BC15i.n. and BC15i.d., BCG-ChAdOx1.PPE15 administered by the i.n. and i.d. routes, respectively.

The route of administration of ChAdOx1.PPE15 is critical for its protective capacity. Boosting of BCG with ChAdOx1.PPE15 by the intradermal route failed to improve the protection provided by BCG, unlike boosting by the intranasal route of administration (Fig. 7a). Lung parenchyma cells, characterized as CXCR3^hi^ KLRG1^lo^, which can be induced by vaccination and M. tuberculosis infection, have previously been associated with protection (37, 38). To investigate whether ChAdOx1.PPE15 was able to induce these cells, mice were vaccinated with ChAdOx1.PPE15 either via the protective intranasal route of administration or via the nonprotective systemic route of administration. Tetramers for the two immunodominant epitopes (epitopes 1.1/1.2 and 5.8) were used in combination with the intravascular staining technique to discriminate antigen-specific cells in the lung parenchyma from antigen-specific cells in lung vascular cells, using an anti-CD45 antibody (39).

Both i.n. and systemic delivery of the vaccine induced a similar number of tetramer-positive CD4^+^ T cells in the lung. However, i.n. delivery resulted in the majority of tetramer-positive cells being located in the parenchyma (CD45^−^); in contrast, delivery by the systemic route resulted in the majority of tetramer-positive cells being associated with the vasculature (CD45^+^) (Fig. 7b and c). The majority of parenchymal cells displayed the protective CXCR3^+^ KLRG1^−^ phenotype, whereas vascular cells were mainly CX3CR1^+^ KLRG1^+^ (Fig. 7d). The number of tetramer-specific CD8^+^ T cells was lower than the number of tetramer-specific CD4^+^ T cells, but the CD8^+^ T cells followed the same location pattern and had the same phenotype as CD4^+^ T cells (Fig. 7e to g). The majority of tetramer-positive lung parenchymal CD4^+^ and CD8^+^ CXCR3^+^ cells also expressed programmed cell death 1 (PD1), a marker generally coexpressed by CXCR3^+^ lung parenchyma cells (37), after i.n. administration of BCG-ChAdOx1.PPE15 (Fig. 7h and i). In contrast, there was no PD1 expression in mice vaccinated i.d. with either BCG or BCG-ChAdOx1.PPE15 (Fig. 7h and i). When the experiment was repeated without a BCG prime, the numbers of CD4^+^ CXCR3^+^ KLRG1^−^ cells were much lower in mice vaccinated with ChAdOx1.PPE15 i.n. than in mice vaccinated with BCG-ChAdOx1.PPE15 i.n., whereas the reverse was true for CD8^+^ CXCR3^+^ KLRG1^−^ cells in the parenchyma (Fig. S6).

These data indicate that the intranasal delivery of ChAdOx1.PPE15 is able to induce antigen-specific CD4^+^ and CD8^+^ cells with a characteristic protective phenotype and that BCG priming affects the ratio of CD4^+^ cells to CD8^+^ cells with this phenotype.

## DISCUSSION

Selecting protective antigens for inclusion in vaccines is a critical step in subunit vaccine development. This is complicated by the lack of immune correlates of protection as well as the absence of a human challenge model. As a result, efficacy in the murine model is a necessary first step in the TB vaccine development pathway. In this study, we screened mycobacterial antigens shown to be strongly and commonly recognized by M. tuberculosis-infected individuals (19, 20) and found that at least one antigen consistently satisfied all criteria of efficacy and immunogenicity. Our findings provide support for this antigen selection process.

Each of the human antigens tested was able to induce robust CD8^+^ T cell responses in active and latently infected individuals (20) and was subsequently confirmed to induce CD4^+^ T cell responses. The following are among the advantages of human recognition as an essential criterion in our antigen selection process: (i) antigens recognized during the course of human M. tuberculosis infection are ideal candidates for human vaccine development, (ii) the antigens are recognized by individuals with active and latent infection; therefore, a vaccine designed to contain these antigens can be used as a pre- and postexposure vaccine; and (iii) candidate antigens are known to also induce CD8^+^ T cells, which play an important role in protection but are somewhat neglected (24, 25, 40).

All four antigens selected were also well recognized during the course of M. tuberculosis infection in mice (see Fig. S1 in the supplemental material), but only two, PPE15 and PPE51, protected against M. tuberculosis when they were used as vaccines (Fig. 2 and 3). Combining these two antigens in one vaccination did not result in an additive protective effect, however (Fig. 3). This could be due to a limitation of our infection setup. We chose an infection dose of 50 to 100 CFU, a dose often used when testing vaccine candidates for efficacy, as 100% of the animals get infected (41–44). After infection, there is a rapid accumulation of mycobacteria in the lungs before the adaptive immune response gets activated (45). However, if this dose is overwhelming the mouse lungs with mycobacteria, there may be a limit to the detectable protective effect in this model (45). Repetition of the challenge experiments with a lower bacterial dose might overcome this, although a lower challenge dose may give more variable results (46). Even though the empty vector was not included in our experiments, the lack of consistent protection by ChAdOx1.PE3 and ChAdOx1.PE12 means that it is unlikely that there is a protective nonspecific effect caused by the vector.

In an attempt to identify immune correlates of protection, epitope mapping experiments were performed in three mouse strains vaccinated with the protective antigens (ChAdOx1.PPE15 and ChAdOx1.PPE51) and the nonprotective antigens (ChAdOx1.PE3 and ChAdOx1.PE12). Some patterns in immune recognition of the protective antigens emerged. Strong immune responses to PPE15 and PPE51 were much more focused to certain regions of the antigen, unlike PE3, which was recognized throughout the whole length, and PE12, which was weakly recognized (Fig. 4 and Fig. S4). PPE15 was the only antigen that was significantly protective in all three challenge experiments performed (Fig. 2, 3, and 5). Interestingly, unlike the other antigens, PPE15's immunodominant epitopes were located at the N-terminal part (Fig. 4a and b and Fig. S4a). There are 69 PPE genes in M. tuberculosis, and they share conserved motifs at their N-terminal domains (47, 48). This could mean that some of them share protective epitopes, which may be presented at different stages of M. tuberculosis infection. A vaccine that uses such epitopes could therefore be more successful, as T cells will always be maintained at high numbers and immune escape is unlikely. However, constant antigenic exposure may lead to T cell exhaustion (49). Alignment of PPE15 with other proteins in its family revealed that it is 69% identical to PPE43 (Rv2768c) and 55% identical to PPE44 (Rv2770c). Interestingly, PPE44 has been shown to protect against M. tuberculosis infection in C57BL/6 mice when administered as a DNA and a recombinant protein with adjuvant (50). In agreement with this, although ChAdOx1.PPE15 failed to improve the efficacy of BCG in BALB/c mice, it did improve the efficacy of BCG in C57BL/6 mice (Fig. 5), potentially indicating the presence of protective epitopes. The possibility that C57BL/6 mouse epitopes are better presented during M. tuberculosis infection was ruled out, as we demonstrated that immunodominant epitopes from both mouse strains were recognized during infection (Fig. 6). Further work is needed to identify which epitope(s) is protective and why and whether other antigens in the PE/PPE family share similar epitopes. Importantly, i.n. ChAdOx1.PPE15 was able to induce lung parenchymal CD4^+^ and CD8^+^ T cells with a protective CXCR3^+^ KLRG1^−^ PD1^+^ phenotype (Fig. 7d [left], g [left], h, and i). This is in contrast to the finding obtained with systemic delivery of the same vaccine, which mainly induced KLRG1^+^ CX3CR1^+^ cells in the vasculature (Fig. 7d [right] and g [right]). Unlike CXCR3^+^ KLRG1^−^ PD1^+^ cells, KLRG1^+^ CX3CR1^+^ cells are highly differentiated and cannot migrate to infected tissue in the lung parenchyma (37). Adoptive transfer experiments are needed to confirm the protective potential of these vaccine-induced cells. Interestingly, a BCG priming immunization seems to result in a higher number of CD4^+^ cells, whereas ChAdOx1.PPE15 given without a prior BCG prime mainly induces a higher CD8^+^ T cell response in the parenchyma. Both ChAdOx1.PPE15 and BCG-ChAdOx1.PPE15 were able to provide protection, unlike their respective control groups, indicating that both CD4^+^ and CD8^+^ cells might play a role in protection. Adoptive transfer experiments would allow the identification of the precise effect of each population. The protective efficacy of PPE15, delivered as a recombinant simian adenoviral vector, will now be assessed in more stringent preclinical models of M. tuberculosis infection, such as in guinea pig and nonhuman primate models.

Both PPE15 and PPE51 are novel mycobacterial antigens, and this is the first report of their potential as vaccine candidates. Other studies showed that both mycobacterial antigens might have important functions. Analysis of the sequence variation of 69 PPE genes among M. tuberculosis complex isolates showed that PPE51 was the only gene that showed no variation (51). Recent evidence showed that PPE51 has a high probability of being associated with the mycobacterial membrane (52). The use of such a conserved antigen could be advantageous, since a vaccine that includes PPE51 might be more effective in a clinical setting with a wide range of M. tuberculosis strains (53). On the contrary, host immune responses to this antigen might be beneficial to the bacterium (9). Similarly, a recent study suggested that PPE15 is involved in the accumulation of triacylglycerol lipid droplets associated with dormancy (54). PPE15 forms part of a set of genes that make up the Esx-5a secretion system of M. tuberculosis. Esx-5a is believed to have been generated by the duplication event of genes encoding two ESAT-6-like proteins and the flanking PE/PPE protein genes; the *esxI*, *esxJ*, *ppe15*, and *pe8* genes form the Esx-5a secretion system (55). Both EsxI and EsxJ were found in the culture filtrates of M. tuberculosis (56), indicating that PPE15 might also be secreted. Interestingly, just like with PPE15, which induced CD8^+^ T cell epitopes in humans, CD8^+^ T cell epitopes were also identified in EsxJ, which was also recognized in patients with active TB disease and individuals with latent M. tuberculosis infection (57, 58).

In conclusion, due to the large number of mycobacterial antigens as well as the lack of immune correlates of protection, selecting an antigen to include in a subunit vaccine presents a rate-limiting step in TB vaccine development. In this study, we followed a selection strategy based primarily on human recognition, expression by BCG, and recognition by cells from M. tuberculosis-infected mice. We suggest that human recognition should be the first step when selecting antigens for human vaccine development. Even though the relevance of the mouse model to human M. tuberculosis infection is unclear, evaluation in the murine model is a necessary step before a vaccine can proceed to later stages of development. For this reason, all selected antigens had to be recognized by infected mouse cells. Finally, after the MVA85A efficacy trial, a vaccine that also induces CD8^+^ T cell responses might be able to improve vaccines that are biased to CD4^+^ T cell responses. Using this approach, two of the antigens conferred significant protection against challenge, with the PPE15 antigen being a very promising vaccine candidate that merits further evaluation. This antigen selection process has the potential to identify more promising M. tuberculosis antigens that could be incorporated into candidate TB subunit vaccines.

## MATERIALS AND METHODS

### Viral vector generation.

Generation of ChAdOx1.PPE15, ChAdOx1.PPE51, ChAdOx1.PE3, and ChAdOx1.PE12 was performed using the Gateway technology (Thermo Fisher Scientific, UK). The four antigens from M. tuberculosis were first cloned into a Gateway entry vector and then recombined into a ChAdOx1 destination plasmid from which E1 and E3 were deleted (29). The viruses were propagated in human embryonic kidney 293 (HEK293) cells and purified by CsCl gradient ultracentrifugation.

The vector carried each antigen under the control of the human cytomegalovirus immediate early promoter, and the antigen was expressed in frame with a signal peptide from the human tissue plasminogen activator for increased immunogenicity (59). All antigens were codon optimized for mammalian expression. The development of ChAdOx1.85A has been previously described (29, 60).

### Mice and immunizations.

Six- to 8-week-old female CB6F1/Crl mice were purchased from Charles River, UK, and C57BL/6 and BALB/c mice were from Harlan, UK. All procedures were performed in accordance with the UK Animals (Scientific Procedures) Act 1986 under project license number 30/2889, granted by the UK Home Office.

BCG Pasteur (ATCC 35734) was grown in-house in 7H9 broth (Becton Dickinson, UK) containing 0.05% Tween 80 (Sigma-Aldrich, UK) and 10% albumin-dextrose-catalase (Sigma-Aldrich, UK). Mice were vaccinated with 4 × 10^5^ CFU per dose via the intradermal (i.d.) route at the ear at 25 μl per ear.

Mice were vaccinated intranasally (i.n.) or intradermally (i.d.) with 1 × 10^8^ infectious units (ifu) of ChAdOx1.PPE15, ChAdOx1.PPE51, ChAdOx1.PE3, and ChAdOx1.PE12 in a final volume of 50 μl. All virus preparations had similar infectious unit-to-viral particle ratios.

For BCG prime-ChAdOx1 boost experiments, a time interval of 10 weeks was allowed. Mice were either harvested for immune responses or infected with M. tuberculosis 4 weeks after the last ChAdOx1 vaccination.

### Flow cytometry.

Cells were extracted from the lung and spleen. Lungs were perfused with phosphate-buffered saline (PBS; Sigma-Aldrich, UK), chopped into small pieces, and digested in DNase/collagenase (Sigma).

Cells were stimulated with a final concentration of 2 μg/ml of each peptide of Ag85A, PPE51, PPE15, PE3, and PE12 in a pool of 15-mer peptides overlapping by 11 amino acids and spanning the whole antigen sequence (Peptide Protein Research, UK) or with medium only for unstimulated controls. After 2 h of stimulation at 37°C, 1 μl/ml of GolgiPlug (BD Biosciences) was added to each well and the cells were incubated for a further 4 h, followed by incubation overnight at 4°C. On the following day, intracellular staining was performed.

Cells were stained for 10 min with LIVE/DEAD fixable dead cell stain (Invitrogen, UK), followed by surface staining with anti-CD45R/B220, TCRαβ, TCRγδ, CD4, and CD8 (eBioscience). Following permeabilization using Cytofix/Cytoperm (BD Biosciences), the cells were stained intracellularly with anti-IFN-γ, anti-TNF-α, anti-IL-2, and anti-IL-17 (eBioscience).

### Tetramer staining.

I-A(b) PPE15_1–15_ phycoerythrin was provided by the NIH Tetramer Facility (Atlanta, GA, USA), and H-2D^b^ PPE15_192–200_-allophycocyanin was purchased from ProImmune (Oxford, UK). Cells were stained with the I-A(b) tetramer at 4°C for 30 min, after which the cells were washed twice with PBS and then surface stained at 4°C with KLRG1, CXCR3, CX3CR1 (all from BioLegend, UK), and PD1 (eBioscience). Dead cells were excluded as described above.

Samples were run on an LSR II flow cytometer, and the data were analyzed using FlowJo (TreeStar Inc., Ashland, OR, USA) and Spice (version 5.3; NIAID, USA) software.

### Intravascular staining.

CB6F1 mice were injected via the lateral tail vein with 100 μl of anti-CD45.2 (clone 104; eBioscience)-fluorescein isothiocyanate at 25 μg/ml. Two minutes later, the lungs were collected in C-tubes containing digestive medium (RPM1 with collagenase and DNase) and processed using a GentleMACS tissue dissociator (Miltenyi Biotec, UK). The lung tissues were digested for 1 h at 37°C, after which they were forced through a 100-μm-mesh-size filter. The cells were then washed, and red blood cells were lysed, washed, and resuspended before tetramer straining.

### M. tuberculosis challenge experiments.

Mice were challenged using a Biaera AeroMP-controlled nebulizer (Biera Technologies, Hagerstown, MD, USA) contained in a biosafety level 3 Total Containment Oxford Ltd (TCOL) isolator. The animals were loaded in nose-only restrainers and exposed to aerosolized M. tuberculosis Erdman K01 (TMC107; BEI Resources, Manassas, VA, USA), prepared at 1 × 10^6^ CFU/ml in the nebulizer. The program was run for 10 min (plus a 5-min purge) at an airflow rate of 12 liters/min and a pressure of 20 lb/in^2^ gauge. Mice were infected with 50 to 100 CFU, which was verified at 24 h after challenge with two mice per experiment.

### Quantification of numbers of CFU.

At 4 weeks postchallenge, the lungs and spleens of the mice were harvested for mycobacterial quantification. Organs were placed in reinforced homogenization tubes (Stretton Scientific, UK) containing 1 ml of PBS and homogenized using a Precellys 24 homogenizer (Stretton Scientific) at 5,500 rpm for 20 s. Dilutions were prepared in PBS and plated on Middlebrook 7H11 (Becton Dickinson, UK) containing glycerol (Sigma-Aldrich) and oleic acid-albumin-dextrose-catalase (BD Diagnostic Systems) according to the manufacturer's instructions. The plates were incubated at 37°C, and counts were obtained 3 weeks later.

### Statistical analyses and presentation.

Statistical analysis was conducted and graphs were generated using GraphPad Prism (version 5) software. Analysis of two data sets was performed using the Mann-Whitney or Kruskal-Wallis test (nonparametric) or one-way analysis of variance followed by *post hoc* tests for comparing three or more groups.

## Supplementary Material

Supplemental material
