# Supplementary material for: Identification and Evaluation of Novel Protective Antigens for the Development of a Candidate Tuberculosis Subunit Vaccine
Source: Infect Immun. 2018 Jun 21;86(7):e00014-18. doi: 10.1128/IAI.00014-18 (PMC6013653; doi:10.1128/IAI.00014-18)
Supplement: Supplemental material [file supp_86_7_e00014-18__index.html]

Supplemental material 

# Identification and Evaluation of Novel Protective Antigens for the Development of a Candidate Tuberculosis Subunit Vaccine

## Supplemental material

- Supplemental file 1 -

  Fig. S1. Antigen recognition by lung and spleen cells from *M. tuberculosis*-infected animals. Fig. S2. Ranking of *M. tuberculosis* antigens based on recognition by spleen and lung cells from *M. tuberculosis*-infected BALB/c and C57BL/6 mice. Fig. S3. CD4+ and CD8+ T cell responses post-ChAdOxl. Fig. S4. Epitope mapping performed after administration of the different ChAdOxl vaccines. Fig. S5. CD4+ or CD8+ restriction of immunodominant PPE15 and PPE51 epitopes. Fig. S6. CD4+ and CD8+ T cells in the lung parenchyma after i.n. or i.d. ChAdOx1.PPE15.

  PDF, 476K
