# Supplementary material for: Identification and Evaluation of Novel Protective Antigens for the Development of a Candidate Tuberculosis Subunit Vaccine
Source: Infect Immun. 2018 Jun 21;86(7):e00014-18. doi: 10.1128/IAI.00014-18 (PMC6013653; doi:10.1128/IAI.00014-18)
Supplement: Supplemental material [file IAI.00014-18_zii999092439s1.pdf]

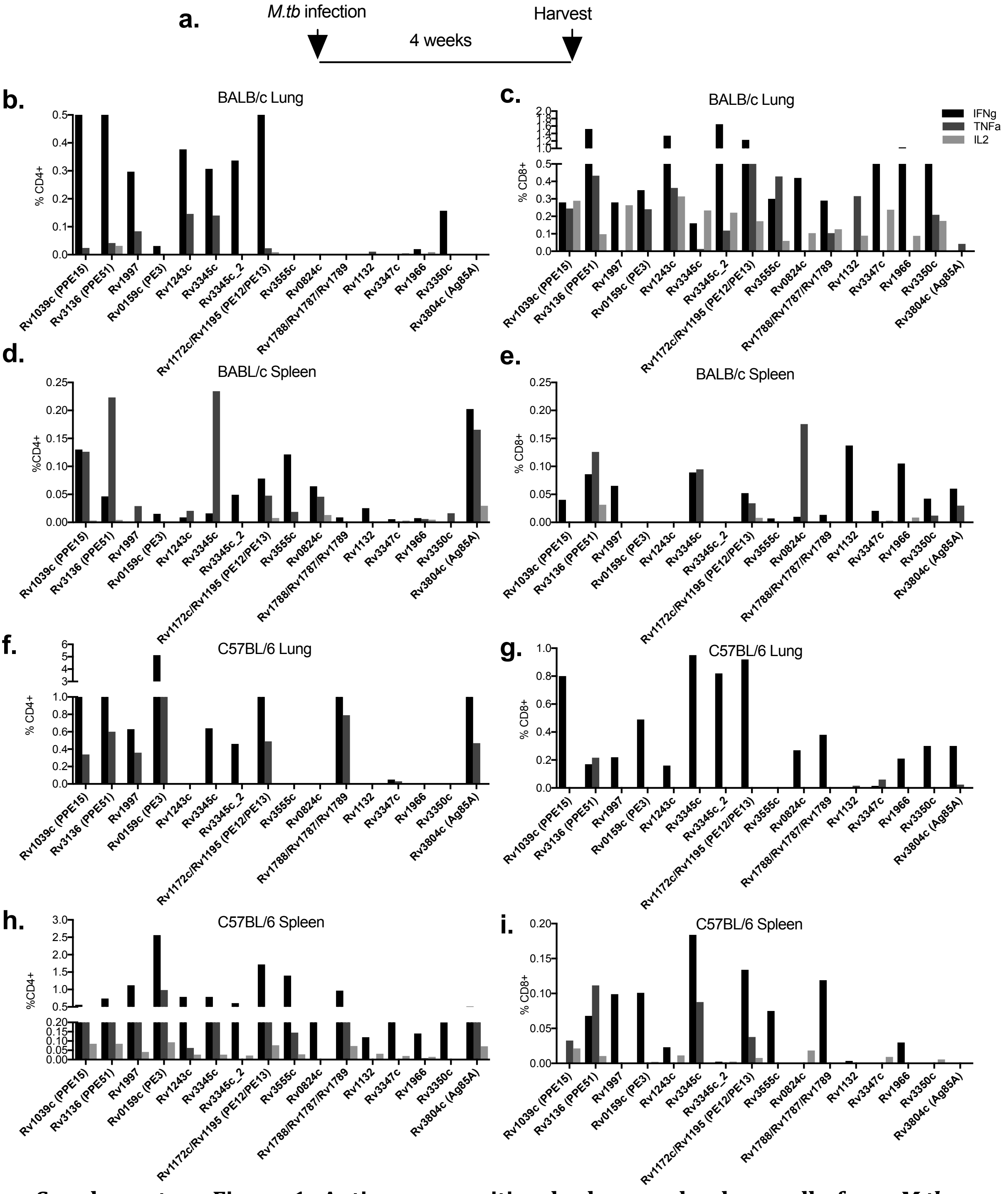

Supplementary Figure 1. Antigen recognition by lung and spleen cells from *M.tb* infected animals.

a. Experimental schema. Lung and spleen cells from infected animals were stimulated with different antigens. The percentage of CD4+ and CD8+ T cells releasing IFNγ/TNFα/IL2 in the **b-c.** lungs and in the **d-e.** spleens of *M.tb* infected BALB/c and in the **f-g.** lungs and **h-i.** spleens of *M.tb* infected C57BL/6 mice 4 weeks post infection is shown. Each bar represents the pooled response from five animals.

Antigen	Balb/c-Lung				C57bl/6-Lung				Balb/c-Spleen				C57BL/6-Spleen				Score	Rank
	CD4 IFNγ	CD8 IFNγ	CD4 γ/α/2	CD8 γ/α/2	CD4 IFNγ	CD8 IFNγ	CD4 γ/α/2	CD8 γ/α/2	CD4 IFNγ	CD8 IFNγ	CD4 γ/α/2	CD8 γ/α/2	CD4 IFNγ	CD8 IFNγ	CD4 γ/α/2	CD8 γ/α/2		
Rv1039c (PPE15)	v	v		v	v	v			v		v		v		v		9/16	**3**
Rv3136 (PPE51)	v	v	v	v	v						v	v	v		v	v	10/16	**2**
Rv1997	v	v			v	v							v		v		6/16	5
Rv0159c (PE3)		v			v	v							v	v	v		6/16	**5**
Rv1243c	v	v		v									v		v		5/16	6
Rv3345c	v				v	v							v	v	v		6/16	5
Rv3345c_2	v	v		v	v	v							v				6/16	5
Rv1172c/ Rv1195 (PE12/ PE13)	v	v	v	v	v	v						v	v	v	v	v	11/16	**1**
Rv3555c		v		v					v		v		v		v		6/16	5
Rv0824c		v				v							v				3/16	8
Rv1788/ Rv1787/ Rv1789		v		v	v	v							v	v	v		7/16	4
Rv1132										v			v				2/16	9
Rv3347c		v			v								v				3/16	8
Rv1966		v				v				v			v		v		5/16	6
Rv3350c		v		v		v							v				4/16	7

Supplementary Figure 2: **Ranking of *M.tb* antigens based on recognition by spleen and lung cells from *M.tb* infected BALB/c and C57BL/6 mice.**

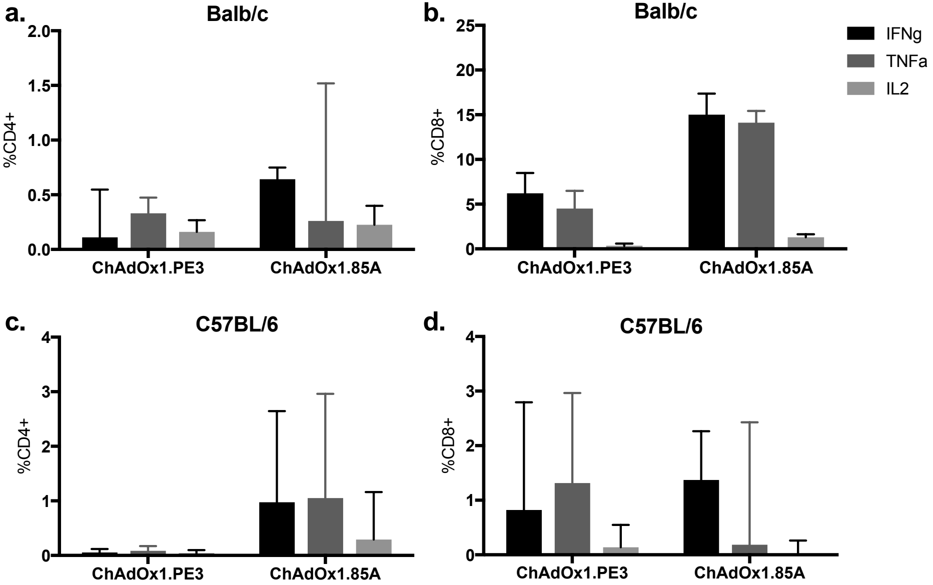

Supplementary Figure 3: CD4+ and CD8+ T cell responses post ChAdOx1. PE3 and Ag85A immunisation. a.-b. Balb/c and **c.-d.** C57BL/6 mice were vaccinated with ChAdOx.PE3 and ChAdOx1.85A intradermally. The percentage of CD4+ and CD8+ T cell secreting cytokines were measured two weeks post vaccination. Bar represents the median value of five mice per group and the line the interquartile range.

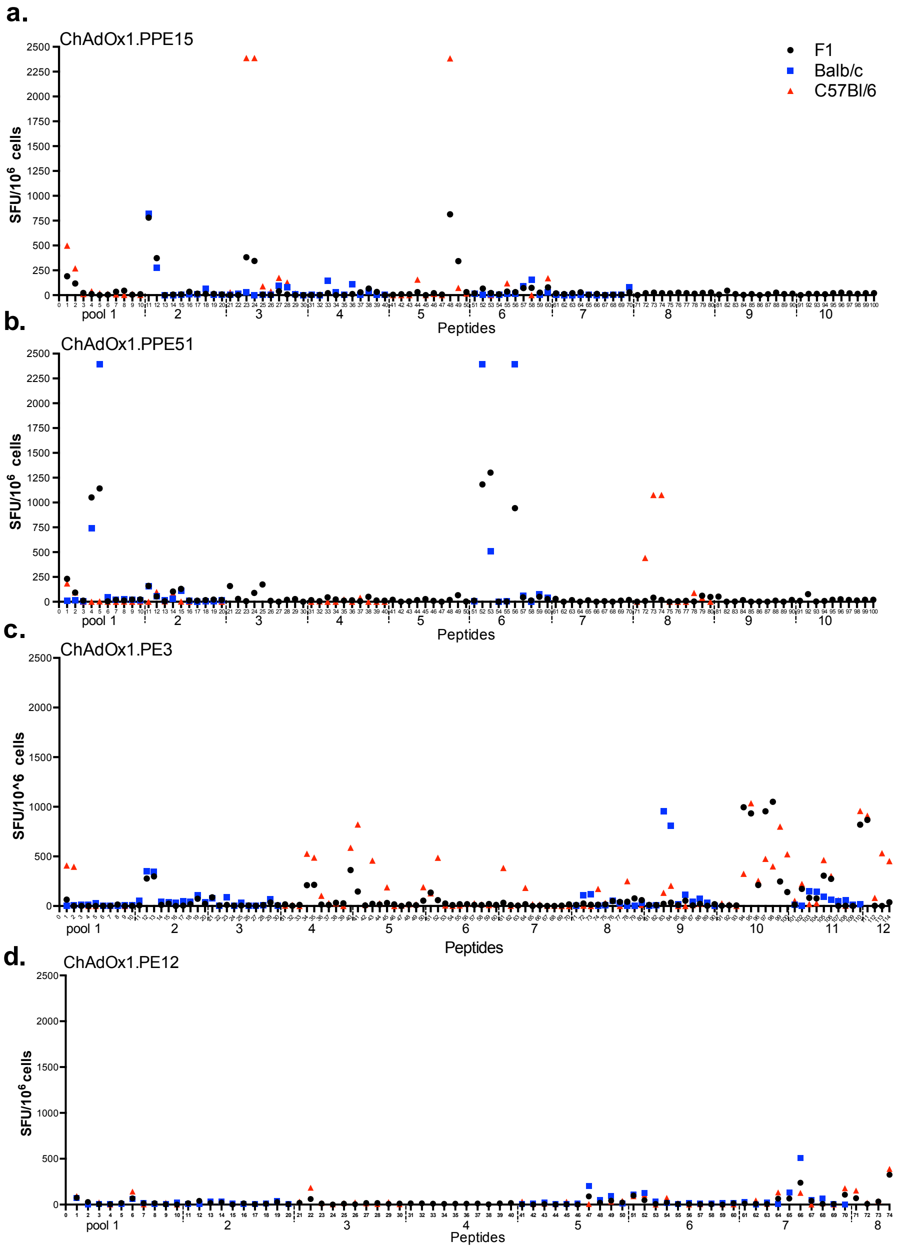

Supplementary Figure 4. Epitope mapping performed after administration of the different ChAdOx1. vaccines. Detailed epitope mapping was performed, by measuring antigen-specific IFNγ responses, two weeks post *i.d.* **a.** ChAdOx1.PPE15, **b.** ChAdOx1.PPE51, **c.** ChAdOx1.PE3 or **d.** ChAdOx1.PE12 vaccination in Balb/c, C57BL/6 and CB6F1 mice. Pooled splenocytes from three vaccinated animals were stimulated with individual 15-mer peptides overlapping by 11 amino acids. Peptide stimulations were performed in duplicate and each dot represents the average.
F1 = black circles, Balb/c = blue squares and C57BL/6 = red triangles.

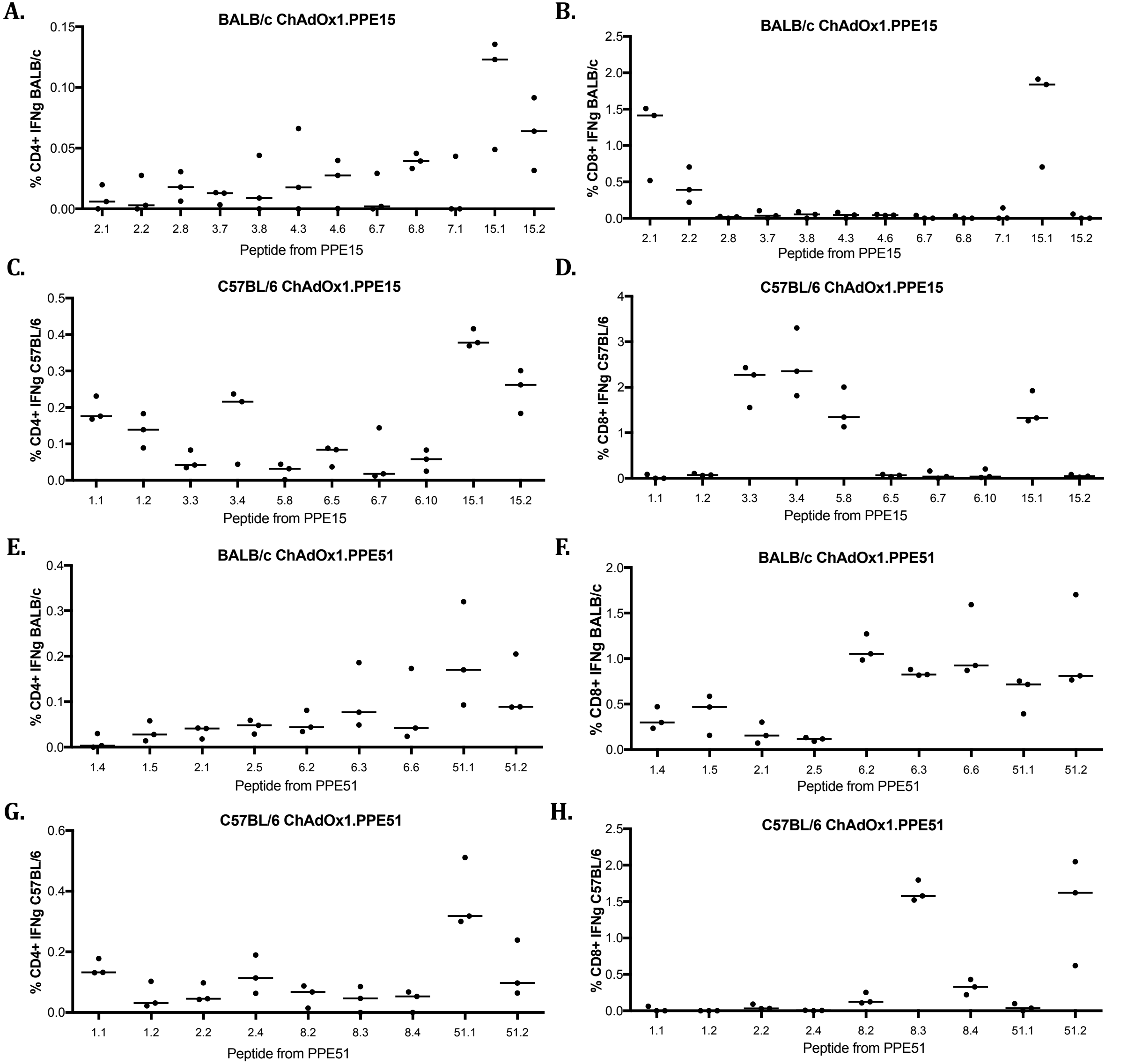

Supplementary Figure 5 CD4+ or CD8+ restriction of immunodominant PPE15 and PPE51 epitopes
Flow cytometry was used to test whether immunodominant peptides were restricted by CD4+ or CD8+ T cells in splenocytes from i.d. vaccinated **A.** BALB/c and **B.** C57BL/5 for **C-D** ChAdOx1.PPE15 and **E-H** for ChAdOx1.PPE51. Each circle represents one animal and the line the median value.

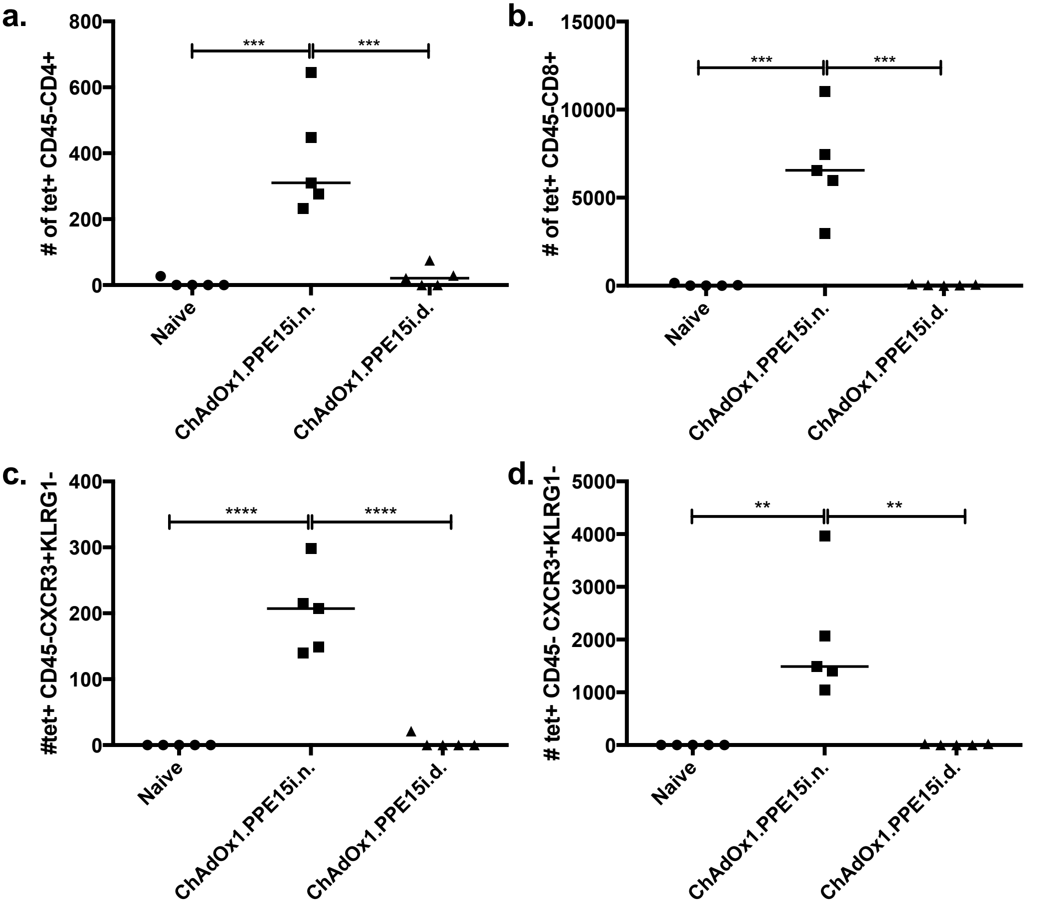

Supplementary Figure 6. CD4+ and CD8+ T cells in the lung parenchyma after i.n. or i.d. ChAdOx1.PPE15. Absolute number of tetramer positive **a.** CD4+ and **b.** CD8+T cells in the lung parenchyma (CD45-). Number of tetramer positive **c.** CD4+ and **d.** CD8+ T cells in the parencyma that are CXCR3+ KLRG1-. Each dot represents one animal and the line the median value for each group. n=5 mice. **p<0.01, ***p<0.001, ****p<0.0001
